# What can “thematic analysis” offer health and wellbeing researchers?

**DOI:** 10.3402/qhw.v9.26152

**Published:** 2014-10-15

**Authors:** Virginia Braun, Victoria Clarke

**Affiliations:** 1 School of Psychology The University of Auckland, Private Bag 92019, Auckland Mail Centre 1142, Auckland, New Zealand. Email: v.braun@auckland.ac.nz; 2 Department of Health and Social SciencesUniversity of the West of England Bristol BS16 1QY, UK

The field of health and wellbeing scholarship has a strong tradition of qualitative research—and rightly so. Qualitative research offers rich and compelling insights into the real worlds, experiences, and perspectives of patients and health care professionals in ways that are completely different to, but also sometimes complimentary to, the knowledge we can obtain through quantitative methods. There is a strong tradition of the use of grounded theory within the field—right from its very origins studying dying in hospital (Glaser & Strauss, 1965)—and this covers the epistemological spectrum from more positivist forms (Glaser, 1992, 1978) through to the constructivist approaches developed by Charmaz (2006) in, for instance, her compelling study of the loss of self in chronic illness (Charmaz, 1983). Similarly, narrative approaches (Riessman, 2007) have been used to provide rich and detailed accounts of the social formations shaping subjective experiences of health and well-being (e.g., Riessman, 2000). Phenomenological and hermeneutic approaches, including the more recently developed interpretative phenomenological analysis (Smith, Flowers, & Larkin, 2009), are similarly regularly used in health and wellbeing research, and they suit it well, oriented as they are to the experiential and interpretative realities of the participants themselves (e.g., Smith & Osborn, 2007).

Thematic analysis (TA) has a less coherent developmental history. It appeared as a “method” in the 1970s but was often variably and inconsistently used. Good specification and guidelines were laid out by Boyatzis (1998) in a key text focused around “coding and theme development” that moved away from the embrace of grounded theory. But “thematic analysis” as a named, claimed, and widely used approach really “took off” within the social and health sciences following the publication of our paper *Using thematic analysis in psychology* in 2006 (Braun & Clarke, 2006; see also Braun & Clarke, 2012, 2013; Braun, Clarke, & Rance, 2014; Braun, Clarke, & Terry, 2014; 2014a, Clarke & Braun, 2014b). The “in psychology” part of the title has been widely disregarded, and the paper is used extensively across a multitude of disciplines, many of which often include a health focus. As tends to be the case when analytic approaches “mature,” different variations of TA have appeared: ours offer a theoretically flexible approach; others (e.g., Boyatzis, 1998; Guest, MacQueen, & Namey, 2012; Joffe, 2011) locate TA implicitly or explicitly within more realist/post-positivist paradigms. They do so through, for instance, advocating the development of coding frames, which facilitate the generation of measures like inter-rater reliability, a concept we find problematic in relation to qualitative research (see Braun & Clarke, 2013). Part of this difference results from the broad framework within which qualitative research is conducted: a “Big Q” *qualitative* framework, or a “small q” more traditional, positivist/quantitative framework (see Kidder & Fine, 1987). Qualitative health and wellbeing researchers will be researching across these research traditions—making TA a method well-suited to the varying needs and requirements of a wide variety of research projects.

Despite the widespread uptake of TA as a formalised method within the qualitative analysis canon, and within health and wellbeing research, we often get emails from researchers saying they have been queried about the validity of TA as a method, or as a method suitable for their particular research project. For instance, we get emails from doctoral students or potential doctoral students, who have been told that “TA isn't sophisticated enough for a doctoral project” or emails from researchers who have been told that TA is only a descriptive or positivist method that requires no interpretative analysis. We get emails from people asking how to respond to reviewer queries on articles submitted for publication, where the validity of TA has been raised. We get so many emails, that we've created a website with answers to many of the questions we get: www.psych.auckland.ac.nz/thematicanalysis.

The queries or critiques often reveal a lack of understanding about the potential of TA, and also about the variability and flexibility of the method. They often seem to assume a realist, descriptive method, and a method that lacks nuance, subtlety, or interpretative depth. This is incorrect. TA *can* be used in a realist or descriptive way, but it is not limited to that. The version of TA we've developed provides a robust, systematic framework for coding qualitative data, and for then using that coding to identify patterns across the dataset in relation to the research question. The questions of what level patterns are sought at, and what interpretations are made of those patterns, are left to the researcher. This is because the techniques are separate from the theoretical orientation of the research. TA can be done poorly, or it can be done within theoretical frameworks you might disagree with, but those are not reasons to reject the whole approach outright.

TA offers a really useful qualitative approach for those doing more *applied* research, which some health research is, or when doing research that steps outside of academia, such as into the policy or practice arenas. TA offers a toolkit for researchers who want to do robust and even sophisticated analyses of qualitative data, but yet focus and present them in a way which is readily accessible to those who aren't part of academic communities. And, as a comparatively easy to learn qualitative analytic approach, without deep theoretical commitments, it works well for research teams where some are more and some are less qualitatively experienced.

Ultimately, choice of analytic approach will depend on a cluster of factors, including what topic the research explores, what the research question is, who conducts the research, what their research experience is, who makes up the intended audience(s) of the research, the theoretical location(s) of the research, the research context, and many others. Some of these are somewhat fluid, some are more fixed. Ultimately, we advocate for an approach to qualitative research which is deliberative, reflective, and thorough. TA provides a tool that can serve these purposes well, but it doesn't serve every purpose. It can be used widely for health and wellbeing research, but it also needs to be used wisely. 



*Virginia Braun*
School of Psychology, The University of Auckland Private Bag 92019, Auckland
Mail Centre 1142 Auckland, New Zealand Email: v.braun@auckland.ac.nz


*Victoria Clarke*
Department of Health and Social Sciences, University of the West of
England Bristol BS16 1QY, UK


